# Editorial: Emotional resilience for wellbeing and employability: the role of learning and training

**DOI:** 10.3389/fpsyg.2024.1379696

**Published:** 2024-03-01

**Authors:** Rasa Smaliukienè, Svajone Bekesiene, Sarka Hoskova-Mayerova

**Affiliations:** ^1^General Jonas Zemaitis Military Academy of Lithuania, Vilnius, Lithuania; ^2^Vilnius Gediminas Technical University - VilniusTech, Vilnius, Lithuania; ^3^University of Defence, Brno, Czechia

**Keywords:** emotional resilience, wellbeing, employability, learning, training, quantitative methods

## Introduction

Developing emotional resilience, the cornerstone of wellbeing in modern human life, plays a key role in the journey to adulthood and a fulfilling career. This essential set of skills plays an important role in the journey to adulthood and is integral to professional success. Emotional resilience not only helps individuals cope with life's transitions but also enables them to overcome the challenges that arise along life's path. It acts as a shield against the stresses of everyday life, promoting wellbeing and productivity. Therefore, learning and training in emotional resilience is becoming increasingly important for individuals and organizations seeking to enhance employee performance.

In recent decades, research on emotions has led to the discovery of foundational components that can be incorporated into curricula tailored to the development of emotion-based knowledge and skills. However, a broad exploration of the emotional landscape can leave obvious gaps in the field of learning and education for emotional resilience, especially in the area of the links between emotional resilience, wellbeing and employability. It is in this context that this theme aims to provide evidence-based insights to broaden the range of learning methodologies and teaching practices that promote emotional resilience.

The Research Topic, entitled “*Emotional Resilience for Wellbeing and Employability: The Role of Learning and Training*,” included 11 papers presenting descriptive and experimental research, as well as methodological suggestions, from an education and training context that focuses on the development of emotional resilience. These 11 contributions provide research-based solutions on how emotional resilience training contributes to employability and wellbeing.

## First field: resources of emotional resilience for employability and wellbeing

When exploring the connection between emotional resilience, employability, and wellbeing, two key categories of resources are essential: personal and organizational. The contributions to this Research Topic have primarily focused on personal resources, such as self-efficacy (BagdŽiuniene et al.), vocational calling (Navickiene and Vasilis Vasiliauskas), grit (Ismail et al.), compassion (Lane et al.), strengths of character, self-regulation, mental agility (Bekesiene et al.), organizational commitment, job embeddedness, and hardiness (Ferreira et al., 2024). These resources are trainable and are the main focus of the research presented in this Research Topic.

Simultaneously, organizational resources, including decent work (Yan et al.), elevated levels of leader-member exchange (LMX) (Song et al.), team cohesion, and peer support (Kanapeckaite and BagdŽiuniene), have been underscored as pivotal elements with significant positive implications for bolstering employees' psychological resilience. In the following sections, important studies will be examined that clarify the reservoir of emotional resilience resources that are relevant to promoting employability and improving wellbeing.

*Organizational resources and self-efficacy*. In research on the emotional resilience of teachers, BagdŽiuniene et al. argues that emotional resilience, defined as the innate ability to navigate, manage, or cope with emotionally challenging situations, is closely linked to teachers' professional and personal resources at work. By examining the combination of teachers' professional environment and personal characteristics, BagdŽiuniene et al. found that teachers' emotional resilience is positively correlated with specific job characteristics such as feedback, autonomy, social support, and professional development opportunities, as well as with teachers' self-efficacy. These two categories of resources—professional and personal—show a positive relationship with teachers' emotional resilience. The cultivation of positive emotions and the ability to maintain emotional equilibrium are important in helping staff to cope with stressful situations and to carry out their daily tasks effectively. Previous studies have already highlighted the contribution of emotional resilience in fostering positive teacher-student relationships (Hagenauer et al., 2015). At an organizational level, the prevalence of positive emotions plays an important role in maintaining a stable workforce and fostering sustainable teaching careers (Lee et al., 2021). Thus, research highlights the positive influence of emotional resilience in enhancing teacher wellbeing. Extending this line of research, BagdŽiuniene et al. found that teachers' emotional resilience mediated the positive relationships between job resources, self-efficacy and teacher wellbeing. Job resources and self-efficacy have a direct positive effect on wellbeing and at the same time act indirectly through emotional resilience as a mediating factor. It is also worth noting the inverse relationship between emotional resilience, job resources, self-efficacy and teachers' intention to leave their current school. Teachers who value the resources provided by their school, have self-confidence and believe in their professional effectiveness are less likely to consider leaving their educational institution.

### Vocational calling and self-efficacy

Focusing on personal resources the study of Navickiene and Vasilis Vasiliauskas on cadets revealed the central role of vocational calling, in shaping psychological resilience. The study examined the moderating effect of vocational calling on the relationship between cadets' resilience and career outcomes, revealing the significant conditional influence of vocational calling on this relationship. Equally important among these personal resources is self-efficacy, which is highlighted in the results of the study by Navickiene and Vasilis Vasiliauskas, as a crucial mediating mechanism that strengthens the link between psychological resilience and professional achievement. As a comprehensive mediator, self-efficacy highlights the pathway through which psychological resilience profoundly influences an individual's professional achievements and potential career outcomes. The findings of this study provide valuable insights into the interplay of personal resources, specifically vocational calling and self-efficacy, and elucidate their role in determining how psychological resilience ultimately influences desired outcomes, such as professional achievements.

### Grit for career advancement

Study Ismail et al. confirms that developing grit, as a protective resilience factor, enables individuals to persist and make consistent progress toward employability. Individuals with grit possess qualities of self-regulation, enabling career mobility through the setting of pragmatic goals and actively seeking new opportunities for professional growth. In addition, grit promotes effort, which in turn cultivates active career resilience, enabling individuals to adapt to challenges and use change for career advancement. Moreover, grit boosts confidence in one's ability to take independent action and demonstrate leadership skills, which is essential for employability.

### Organizational commitment, job embeddedness, and hardiness

According to Ferreira et al. (2024), organizational commitment, job embeddedness and hardiness significantly predict employees' psychological resilience and career adaptability. In analyzing these relationships, Ferreira et al. (2024) found that organizational commitment has the greatest influence when analyzing human resources in global digital mindset and financial services organizations. The other two factors, job embeddedness and hardiness, also contribute but to a lesser extent. This suggests that promoting job engagement is key to increasing employees' resilience and adaptability in the workplace.

### Compassion in professional practice

Adapting positively to challenges in professional life and leading to wellbeing requires more than just emotional resilience. Lane et al. highlight the importance of cultivating compassion in medicine and promoting patient-centered practice. Compassion, defined as an affectionate motivation to reduce the suffering of others, can benefit medical professionals, recipients of compassionate actions, and the medical systems that support them. Lane et al. found that despite the benefits, several factors inhibited the enactment of compassion, including a toxic culture in medical education, disrespect, and time constraints. These factors in organizational culture highlight potential threats to emotional wellbeing in a professional setting. If not managed effectively, these challenges can undermine the ability to sustain compassionate care and effectively manage the demands of the profession. However, it is important to note that compassion is not a fixed trait, but rather something that can be cultivated and grown through experience. This can be achieved in an educational environment.

### The role of decent work

Organizational resources are as well important as personal in improving psychological resilience in the workplace. The Yan et al. study supports this assertion, as it was designed to evaluate the impact of decent work on employee innovation. Decent work is an organizational resource that aims to provide equal opportunities and promote a sense of security and dignity among employees. According to Yan et al. research, decent work can be considered an organizational resource that positively impacts fostering innovative behavior among individuals who practice it. Additionally, it can help mitigate the likelihood of burnout. However, the study shows that the impact of organizational resources on worker burnout and general wellbeing is not always direct, especially when considering multifaceted factors.

### The role of team cohesion and peer support

The interdependence of organizational and personal resources of psychological resilience is found in the study by Kanapeckaite and BagdŽiuniene. The study provide evidence that that team cohesion and peer support in the military have a positive impact on psychological resilience. Additionally, individuals with greater resilience are more committed to the organization and experience higher levels of wellbeing. The results suggest that individual psychological resilience mediates the relationship between team characteristics and commitment and wellbeing (Kanapeckaite and BagdŽiuniene).

Structural equation modeling was used by Ferreira et al. (2024). To facilitate data analysis, BagdŽiuniene et al. and Navickiene and Vasilis Vasiliauskas effectively utilized the PROCESS macro 3.5v, a tool developed by Hayes and Scharkow (2013). Kanapeckaite and BagdŽiuniene tested relationships between variables using the IBM SPSS Statistics 29v and SPSS AMOS 29v software, the smart PLS 3.0 was used by Song et al., and Yan et al. analyzed the data by hierarchical linear model (HLM). Additionally, Bekesiene et al. specifically employed the Decision-making Trial and Evaluation Laboratory (DEMATEL) method to discern relationships among criteria and allocate weight coefficients.

Taken together, these studies highlight the complex and intertwined nature of emotional resilience and its dependence on multiple interactions between personal characteristics and the organizational environment, confirming its essential role in promoting employability and wellbeing in the workplace.

## Second field: training and education for emotional resilience at work

### Organizational impact of training initiatives

In addition to fostering a supportive organizational culture and making use of various resources, organizations can actively support their employees in cultivating emotional resilience through tailored training and education programmes aimed at strengthening relevant competencies. In addition to fostering a conducive organizational culture and leveraging various resources, organizations can actively support their employees in cultivating emotional resilience through tailored training and educational programs aimed at strengthening relevant competencies. This can be seen in the randomized controlled trial evaluation conducted by Plant et al., which highlights the potential impact of organizational training initiatives. The significant interaction effect between treatment group and time period was identified by employing a mixed MANOVA analysis (Plant et al.). The study found that vitality training, which uses behavior-change techniques, significantly increased employees' energy levels while reducing stress. Worth noting is the promising implication of these outcomes in positively influencing indicators associated with burnout, evident upon the conclusion of the vitality training experiment.

### Foundations in psychological resilience training in organization

The fundamentals of psychological resilience training could be found in positive psychology theory according to Bekesiene et al.. An individual's ability to effectively adapt to challenging circumstances could be trained, as successful adaptation depends on two main assumptions: the experience of stress and the subsequent positive response of the individual in his or her own best interest. Positive psychology posits numerous determinants of emotional resilience, prompting an investigation into the most important factors. Bekesiene et al. sought to address this inquiry through a reductionist approach to resilience training, identifying key competencies. Recognizing the contextual variability of resilience, the study by Bekesiene et al. focused on the military environment using two groups of experts: those versed in domestic conflicts and those with extensive experience in international military missions. Evaluating the widely acclaimed Master Resilience Training (MRT) programme, which is recognized for its effectiveness in military resilience training, Bekesiene et al. found that although different competencies become more relevant in different stress contexts, the composition of the main competencies that contribute to the resilience of a soldier remains consistent. The entire program of emotional resilience can be focused on three main competencies and their development: strengths of character, self-regulation, and mental agility.

### Organizational resources for psychological resilience

Song et al. discovered that orientation training, when combined with high levels of leader-member exchange (LMX), can function as an organizational resource that directly and positively affects newcomers' Psychological Capital (PsyCap). PsyCap refers to an individual's positive developmental state and is a personal resource for psychological resilience. The research indicates that organizational resources, such as orientation training, have a significant impact on personal resources. The study conducted by Song et al. demonstrates a predictive relationship between orientation training, an organizational resource, and an individual's work engagement, a personal resource. Additionally, the research presents conclusive evidence that emphasizes the interconnectedness and simultaneous development of these two types of psychological resilience resources, highlighting their interdependence.

These research findings highlight the significant impact that tailored training programmes and educational initiatives can have on improving emotional resilience in organizations. Such initiatives not only strengthen individual competencies, but also provide a solid foundation for sustained well-being and engagement in the workplace.

## Further perspectives

Based on research findings resented in this Research Topicseveral future perspectives in emotional resilience learning and training for wellbeing and employability could be drawn.

### Research into effective intervention strategies

The research presented in this Research Topicdemonstrates that interventions promoting the development of psychological resilience yield positive results. There is evidence that vitality training, which employs behavioral change techniques, significantly increases employees' energy levels while reducing stress (Plant et al.). Additionally, orientation training, when combined with high levels of leader-member exchange (LMX), has a positive effect on individual work engagement and employees' psychological capital (Song et al.). In the future, it would be valuable to explore mentoring models and interventions that promote emotional resilience, in addition to curricula. This should take into account contextual and institutional barriers. Lane et al.'s study on compassion in medicine and the promotion of patient-centered practice highlights potential threats to emotional wellbeing in a professional setting due to organizational culture. Further research could explore the impact of institutional support on the development of psychological resilience. Specifically, examining the impact of institutional policies, resources, and support systems on staff would be useful. Furthermore, it is crucial to investigate the systemic modifications required to foster psychological resilience in employees. This could significantly enhance the emotional wellbeing of employees.

### Repeated measures and longitudinal studies

One limitation observed in most of the studies presented in this Research Topicis their cross-sectional nature, where data were collected at a single point in time. To provide psychological resilience training research, repeated measures and longitudinal studies are of great value, as illustrated by the studies of Plant et al. and Song et al.. These studies illustrate the benefits of using repeated measures and provide a clearer understanding of the evolving landscape of emotional resilience development. Encouraging the wider use of these methods in future research would provide a more comprehensive view, allowing changes to be tracked over time. In addition, their use facilitates the establishment of precise causal relationships between variables, while mitigating the biases often associated with cross-sectional data.

Expanding research across different fields, national cultures, and geographic regions is essential to enhance the generalizability of findings concerning emotional resilience training for wellbeing and employability. The research outcomes highlighted in this Research Topicprovide insightful perspectives on resilience in various domains, encompassing the resilience of teachers (BagdŽiuniene et al.), military personnel and military applicants (Bekesiene et al.; Navickiene and Vasilis Vasiliauskas; Sedlačík et al.; Kanapeckaite and BagdŽiuniene), medical practitioners (Lane et al.), as well as knowledge workers (Ferreira et al., 2024; Yan et al.). These studies emphasize the distinctiveness of each domain and the various personal and organizational resources that contribute to building psychological resilience. In this Research Topic, Plant et al. and Ismail et al. present research outcomes from multiple fields of activity, providing a more comprehensive understanding of resilience training across varied settings. The combined insights from these studies highlight the importance of acknowledging different emotional models and subtle nuances within resilience education in different contexts that could be extended in further research.

## Author contributions

RS: Writing – original draft, Writing – review & editing. SB: Writing – original draft, Writing – review & editing. SH-M: Writing – original draft, Writing – review & editing.
